# An Open Letter

**Published:** 1893-04

**Authors:** J. R. Haynes

**Affiliations:** Indianapolis, Ind.


### AN OPEN LETTER—POSTSCRIPT.

INDIANAPOLIS, IND., April 18th, 1893.

THE EDITOR OF THE HOMŒOPATHIC PHYSICIAN :

In my open letter, published in the March number at page 188, I stated that Dr. Dunham died on the morning of February 18th, 1877, several years before Dr. Conrad Wesselhœft's translation was published, and therefore Dr. Dunham could not have examined it (see bottom of page 190) ; but upon looking over some old letters from Dr. Hering and Dr. Ad. Lippe I find that they had copies of it in 1876, therefore I wish that you would make this correction and in the next number if possible, as I do not desire to state anything but fact.

I formed my statement upon the imprint in the copy that I have which was ordered as soon as the first advertisements made their appearance of the translation and that was the copy that I received. This being the case then, Dr. Dunham *could* have examined it (the translation) and given his opinion.

Very respectfully,

J. R. HAYNES.

